# Immunohistochemical Characteristics of IgG4-Related Tubulointerstitial Nephritis: Detailed Analysis of 20 Japanese Cases

**DOI:** 10.1155/2012/609795

**Published:** 2012-07-31

**Authors:** Mitsuhiro Kawano, Ichiro Mizushima, Yutaka Yamaguchi, Naofumi Imai, Hitoshi Nakashima, Shinichi Nishi, Satoshi Hisano, Nobuaki Yamanaka, Motohisa Yamamoto, Hiroki Takahashi, Hisanori Umehara, Takao Saito, Takako Saeki

**Affiliations:** ^1^Division of Rheumatology, Department of Internal Medicine, Kanazawa University Hospital, Kanazawa, Ishikawa 920-8641, Japan; ^2^Yamaguchi's Pathology Laboratory, Matsudo, Chiba 270-2231, Japan; ^3^Division of Clinical Nephrology and Rheumatology, Niigata University Graduate School of Medical and Dental Sciences, Niigata 951-8510, Japan; ^4^Division of Nephrology and Rheumatology, Department of Internal Medicine, Faculty of Medicine, Fukuoka University, Fukuoka 814-0180, Japan; ^5^Division of Nephrology and Kidney Center, Kobe University Graduate School of Medicine, Kobe 650-0017, Japan; ^6^Department of Pathology, Faculty of Medicine, Fukuoka University, Fukuoka 814-0180, Japan; ^7^Tokyo Kidney Research Institute, Tokyo 113-0023, Japan; ^8^First Department of Internal Medicine, Sapporo Medical University School of Medicine, Sapporo 060-8543, Japan; ^9^Department of Hematology and Immunology, Kanazawa Medical University, Kanazawa 920-0293, Ishikawa, Japan; ^10^Department of Internal Medicine, Nagaoka Red Cross Hospital, Nagaoka 940-2085, Japan

## Abstract

Although tubulointerstitial nephritis with IgG4+ plasma cell (PC) infiltration is a hallmark of IgG4-related kidney disease (IgG4-RKD), only a few studies are available about the minimum number of IgG4+ PC needed for diagnosis along with IgG4+/IgG+ PC ratio in the kidney. In addition, the significance of the deposition of IgG or complement as a reflection of humoral immunity involvement is still uncertain. In this study, we analyzed 20 Japanese patients with IgG4-RKD to evaluate the number of IgG4+ PCs along with IgG4+/IgG+ PC ratio and involvement of humoral immunity. The average number of IgG4+ PCs was 43.8/hpf and the average IgG4+/IgG+ or IgG4+/CD138+ ratio was 53%. IgG and C3 granular deposits on the tubular basement membrane (TBM) were detected by immunofluorescence microscopy in 13% and 47% of patients, respectively. Nine patients had a variety of glomerular lesions, and 7 of them had immunoglobulin or complement deposition in the glomerulus. In conclusion, we confirmed that infiltrating IgG4+ PCs > 10/hpf and/or IgG4/IgG (CD138)+ PCs > 40% was appropriate as an item of the diagnostic criteria for IgG4-RKD. A relatively high frequency of diverse glomerular lesions with immunoglobulin or complement deposits and deposits in TBM may be evidence of immune complex involvement in IgG4-related disease.

## 1. Introduction

The main histopathological finding in the kidney of IgG4-RD is tubulointerstitial nephritis (TIN) [1–3], which may result in renal failure [4]. IgG4-related TIN is composed of dense lymphoplasmacytic infiltrates with fibrosis and copious IgG4+ plasma cell infiltration, which are common features shared by other involved organs [5], and these common pathologic features in the kidney have clearly been described by previous studies [1–3]. However, the minimum number of IgG4+ plasma cells needed for diagnosis has been differently reported in each affected organ [6–9], and only a few studies are available about the actual number of IgG4+ plasma cells evaluated along with IgG4+/IgG+ plasma cell ratio in IgG4-related kidney disease (IgG4-RKD) [2].

 In addition to this issue, case reports or case series of a variety of glomerular disease concurrent with TIN have been accumulated [10–26]. These glomerular lesions are frequently accompanied by immunoglobulin or complement deposits suggesting that immune complexes might be involved in the pathogenesis of some cases with IgG4-RKD [2, 3]. However, the significance of these glomerular lesions as a reflection of humoral immunity involvement is still uncertain, and whether these glomerular lesions represent some IgG4-related kidney lesions with common etiopathological background or unrelated lesions merely concurrent with IgG4-TIN is still controversial.

In this study, we analyzed 20 Japanese patients with IgG4-RKD that were collected in our previous study aimed at establishing diagnostic criteria for IgG4-RKD [27], to address these pathological issues about the number of IgG4+ plasma cells along with IgG4+/IgG+ plasma cell ratio and involvement of humoral immunity in Japanese IgG4-RKD patients. 

## 2. Methods

### 2.1. Patients

 Between 2004 and 2011, we found 41 patients with IgG4-RKD in Kanazawa University Hospital, Nagaoka Red Cross Hospital, Niigata University Hospital, Sapporo Medical University Hospital, and Fukuoka University Hospital, of whom 28 underwent renal biopsy. In the remaining 13 patients with IgG4-RKD without renal biopsy, 4 had only pelvic lesion and 9 had typical radiologic findings such as multiple low-density lesions on enhanced CT, high serum IgG4 levels, and other organ involvement with biopsy proven IgG4+ plasma cell infiltration. In addition, these 9 patients had radiographic improvement after successful corticosteroid treatment. Of these 28 patients, 20 who received renal needle biopsy were included in this study because they had sufficient data to determine the number of IgG4-positive cells, IgG4/IgG or IgG4/CD138 ratio, and immunofluorescence microscopy or electron microscopy. Five patients with glomerular lesions (2 Henoch-Schönlein purpura [28, 29]; 2 membranous glomerulonephritis [4, 30]; 1 membranoproliferative glomerulonephritis [23]) were reported as case reports previously. Ten patients with crescentic glomerulonephritis or antineutrophil cytoplasmic antibodies (ANCA) associated vasculitis (1 Churg-Strauss syndrome; 1 Wegener's granulomatosis; 4 microscopic polyangiitis; 4 renal limited ANCA vasculitis) were also included in the study of infiltrating IgG4+ plasma cells as a control because IgG4+ plasma cell infiltration in some patients with ANCA associated vasculitis has been shown in previous studies [2, 31, 32]. Written informed consent for use of all data and samples was obtained from each patient. The diagnosis of IgG4-RKD was made based on the histopathologic findings of one or more organs, characteristic diagnostic imaging findings, elevated serum IgG4 levels, and other organ involvement typical for IgG4-RD. This study was approved by each institutional ethics board and the ethics board of the Japanese Society of Nephrology. The research was conducted in compliance with the Declaration of Helsinki.

### 2.2. Clinical Features

 The clinical picture including allergic symptoms and those resulting from other organ involvement of IgG4-RD was noted. Serum IgG, IgG4, IgE, complement, and creatinine levels were obtained from the clinical data file. Urinary abnormalities including proteinuria, hematuria, and casturia were collected. 

### 2.3. Imaging

 Computed tomography (CT) with or without enhancement with contrast medium was performed before corticosteroid therapy to make the diagnosis of kidney involvement. Other modalities including gallium scintigraphy, magnetic resonance imaging, and fluorodeoxyglucose positron emission tomography were also employed to identify renal and extra-renal lesions.

### 2.4. Histology and Immunostaining

 Bouin's fluid-fixed or formalin-fixed and paraffin-embedded renal specimens of patients with IgG4-RKD were analyzed, and tubulointerstitial nephritis with or without glomerular lesions was evaluated. These specimens were stained with hematoxylin and eosin (HE), periodic acid-Schiff (PAS), periodic acid methenamine silver (PAM), and Masson's trichrome for light microscopy (LM). Immunofluorescence microscopy was performed against IgG, IgA, IgM, C3, C1q, and fibrinogen. Immunostaining for infiltrating plasma cells was performed using mouse monoclonal antibody against human IgG4 (Zymed Laboratory, San Francisco, CA, USA, or The Binding Site, Birmingham, UK), antihuman IgG (Dako, Glostrup, Denmark), and/or antihuman CD138 (AbD serotec, Oxford, UK). IgG4+ plasma cells were counted in five different high power fields (hpf) (×400 magnification with an eyepiece with a field number of 22) with intensive infiltration, and the average IgG4+ plasma cell count was calculated. Average of IgG4+/IgG+ or IgG4+/CD138+ plasma cell ratio of at least two different hpf (2–5 hpf) was calculated. 

### 2.5. Statistical Analysis

 Mann-Whitney *U* test or Fisher's exact probability test was employed for the statistical analyses. A value of <0.05 was considered statistically significant. 

## 3. Results

### 3.1. Clinical and Laboratory Features

 The patients were 18 men and 2 women with an average age 64 years (range: 55 to 83).  Table 1 shows clinical and laboratory features of the patients with IgG4-related TIN. Six patients had elevated serum creatinine levels (>2 mg/dL). The mean serum IgG level was 3479 mg/dL (range 1679–5380 mg/dL), and the mean serum IgG4 level was 923 mg/dL (range 408–1860 mg/dL) with all patients having elevated serum IgG4 levels. Hypocomplementemia was detected in 13 patients. Serum IgE level was evaluated in 11 of 12 patients tested. All patients except one had other organ involvement, and the clinical picture in relation to systemic organ involvement contributed to making the diagnosis of IgG4-RD. Frequently, involved organs were the salivary gland, pancreas, and lung. Twelve patients had sialadenitis, and 7 autoimmune pancreatitis type 1.

### 3.2. Histology and Immunostaining

**Figure 1:**
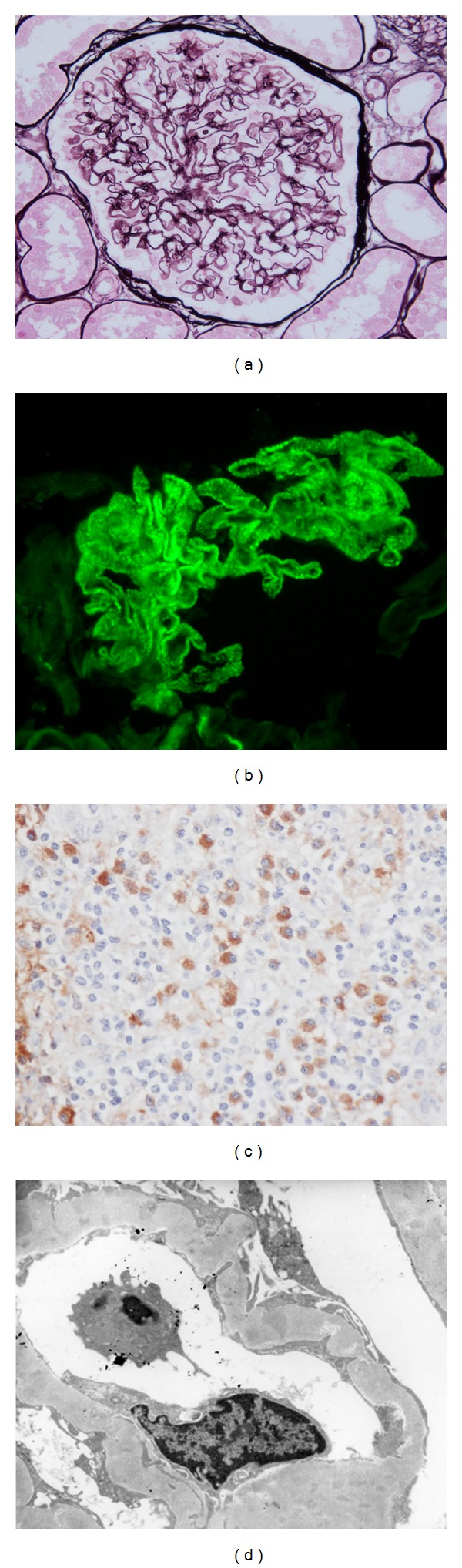
IgG4-related tubulointerstitial nephritis with membranous glomerulonephritis. (a) Periodic acid methenamine silver (PAM) staining reveals spike and bubbling formation (PAM ×400). (b) Immunofluorescence staining for IgG reveals granular deposits along the glomerular capillary walls (×400). (c) Many IgG4+ plasma cells are seen in the interstitium (IgG4 ×400). (d) Electron microscopy (EM) shows subepithelial deposits and variable reabsorption of these deposits with thickened glomerular basement membrane. (Ehrenreich-Churg stage II–IV).

**Figure 2:**
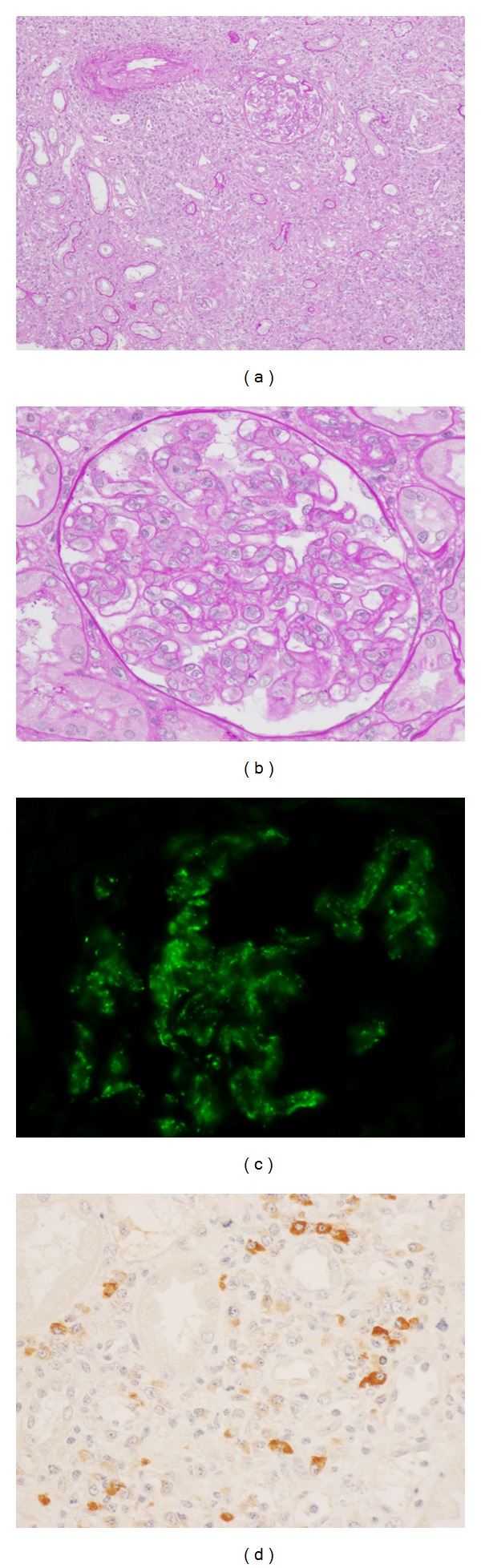
IgG4-related tubulointerstitial nephritis with Henoch-Schönlein purpura nephritis. (a) Periodic acid-Schiff (PAS) staining reveals severe tubulointerstitial nephritis (PAS ×100). (b) Global endocapillary proliferation is evident (PAS ×400). (c) Immunofluorescence staining for C3 reveals mesangial and capillary wall deposits (×400). (d) Many IgG4+ plasma cells are seen in the interstitium (IgG4 ×400).

**Figure 3:**
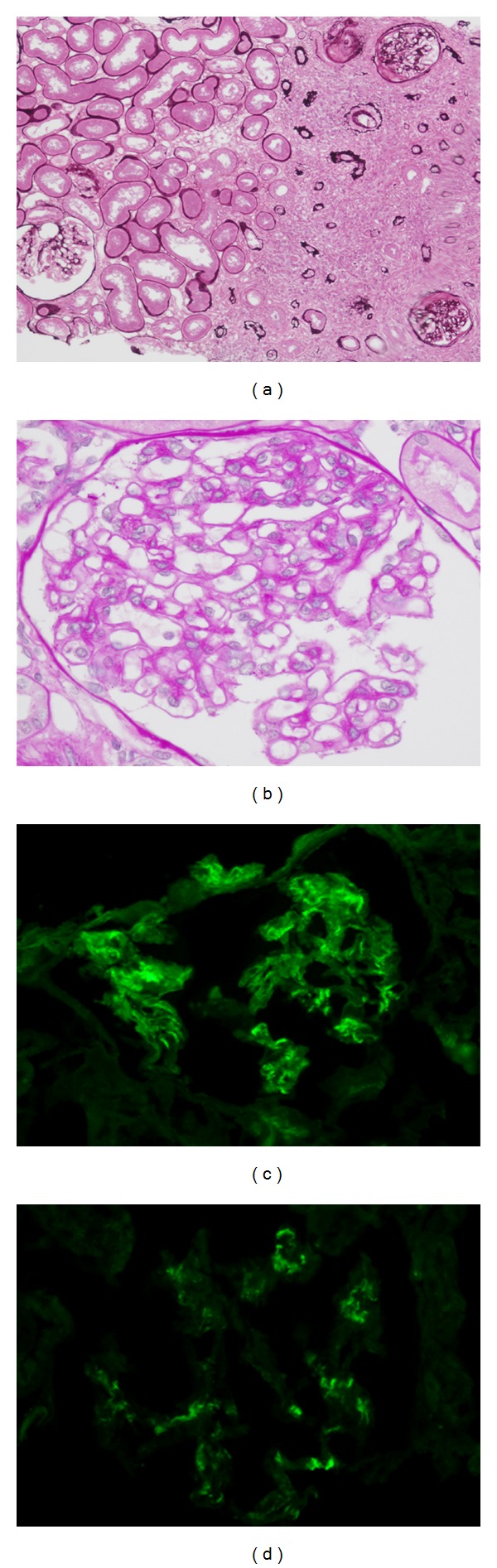
IgG4-related tubulointerstitial nephritis with IgA nephropathy. (a) Periodic acid-Schiff (PAS) staining reveals severe tubulointerstitial nephritis (PAS x100). Regional lesion distribution is evident. (b) Segmental mesangial proliferation is seen (PAS ×400). (c) Immunofluorescence staining for IgA reveals bright mesangial deposits (×400). (d) Immunofluorescence staining for C3 reveals weak mesangial staining for C3 (×400).

Table 2 shows histologic features of 20 patients with IgG4-related TIN. Dense lymphoplasmacytic infiltration with fibrosis in the interstitium was a common feature, but one patient did not have obvious fibrosis. In immunohistochemistry, the average number of IgG4 positive plasma cells was 43.8/hpf (range 10–156/hpf), and average IgG4+/IgG+ or IgG4+/CD138+ ratio was 53% (range 18–90%). All patients fulfilled the histologic part of our diagnostic criteria for IgG4-related kidney disease, namely, infiltrating IgG4-positive plasma cells >10/hpf and/or IgG4/IgG (CD138)-positive plasma cells >40% [27]. IgG and C3 granular deposits on the tubular basement membrane (TBM) were detected by immunofluorescence microscopy in 2 (13%) and 7 (47%) of 15 patients for whom pathological reports about TBM staining were available. Granular C1q deposits on TBM were detected by IF in 2 (13%) of 15 patients. Of these, C3 granular deposits in the tubular basement membranes without accompanying IgG were thought to be a nonspecific feature because of possible production of C3 by tubular epithelial cells. Electron dense deposits were detected by electron microscopy (EM) in 6 (40%) of 15 patients. Glomerular lesions concurred with IgG4-related TIN in 9 patients, in all of whom other immune complex-mediated glomerulopathies such as lupus nephritis, Sjögren's syndrome, and cryoglobulinemia were ruled out by appropriate clinical, biochemical, serological, and other testing. The most frequently observed glomerular lesion was membranous glomerulonephritis, and three patients had this lesion (Figure 1). These patients did not have any mesangial or subendothelial dense deposits suggesting secondary membranous glomerulonephritis such as lupus nephritis. Similarly, they did not have clinical features suggesting secondary forms of membranous glomerulonephritis such as hepatitis B or C. Two patients had Henoch-Schönlein purpura nephritis (Figure 2) with typical purpuric skin lesions, the histopathology of which was composed of typical leukocytoclastic vasculitis with neutrophils and rare IgG4+ plasma cells. In addition, one patient showed IgA positive staining in the skin, while IgA immunostaining was not performed in the other patient. The remaining glomerular lesions were IgA nephropathy (Figure 3), membranoproliferative glomerulonephritis, and focal and segmental endocapillary hypercellularity. 

### 3.3. Comparison between IgG4-Related TIN with and without Glomerular Lesions

Table 3 shows a comparison between IgG4-related TIN with or without glomerular lesions. The mean age of the glomerular lesion positive group (GL group) was higher than that of the glomerular lesion negative group (nonGL group) (73.8 ± 7.2 versus 66.0 ± 7.7 y; *P* < 0.05). Serum C3 levels of the GL group tended to be lower than those of the nonGL group (43 ± 23 versus 70 ± 27), but the difference was not statistically significant. The average number of IgG4 positive plasma cells, average IgG4+/IgG+ or IgG4+/CD138+ ratio, frequency of IgG, C3, C1q, and electron dense deposits on the TBM were not significantly different between the two groups.

### 3.4. IgG4-Positive Plasma-Cell-Rich ANCA-Associated Vasculitis

**Figure 4:**
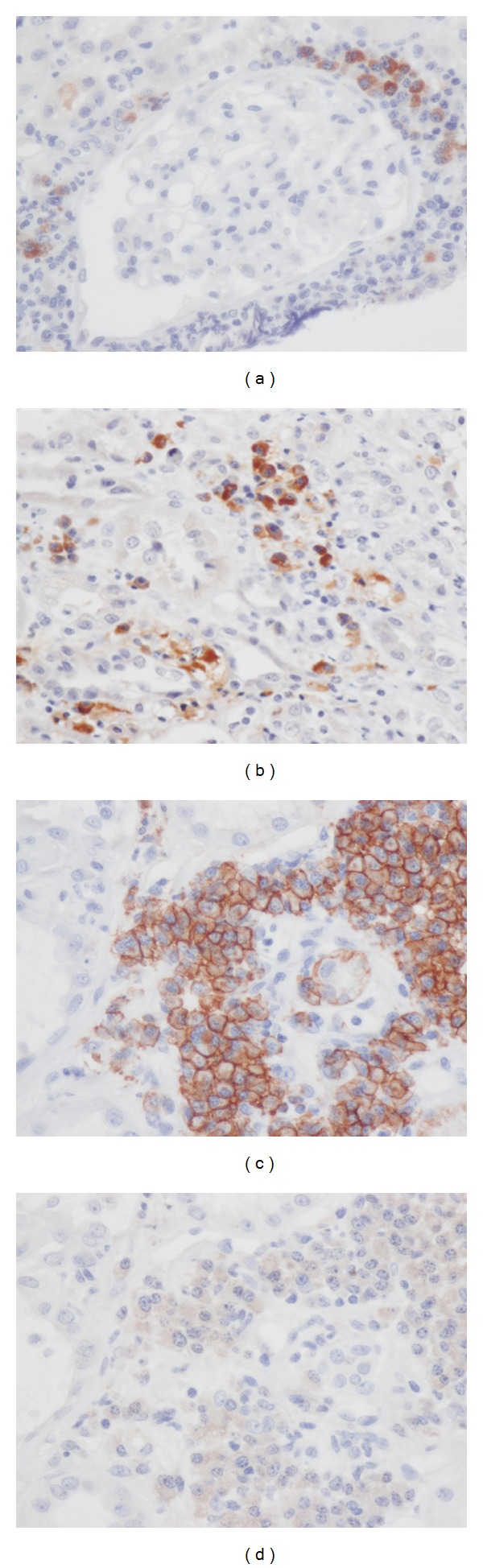
Anti-neutrophil cytoplasmic antibodies (ANCA) associated vasculitis. (a) IgG4+ plasma cells surround a glomerulus (IgG4 immunostaining ×400). (b) Accumulation of many IgG4+ plasma cells is seen in the interstitium (IgG4 immunostaining ×400). (c) Many CD138+ cells are seen in the interstitium (CD138 immunostaining ×400). (d) These plasma cells are IgG4 negative (IgG4 immunostaining x400).

 We analyzed 10 patients with ANCA-associated vasculitis immunohistochemically. Of these, 6 patients had more than 30/hpf plasma cell infiltration in the interstitium. Using IgG4 immunostaining, we found four patients with ANCA-associated vasculitis who fulfilled the immunohistochemical item of the diagnostic criteria of IgG4-related kidney disease (Figures 4(a) and 4(b)).  Table 4 shows a summary of these four patients, all of whom had infiltrating IgG4-positive plasma cells >10/hpf and IgG4/CD138-positive plasma cells >40%. In contrast, in 2 patients only a small part of the infiltrating plasma cells were IgG4 positive (Figures 4(c) and 4(d)). 

## 4. Discussion

 In this study, we showed data about IgG4 positive plasma cell number per high power field (hpf) and IgG4+/IgG+ or IgG4+/CD138+ plasma cell ratios in the kidneys in some Japanese patients with IgG4-RKD. In addition, we compared IgG4-RKD patients with glomerular lesions with those without them clinically. 

 The number of IgG4+ plasma cells varies in affected organs and according to the biopsy method used (percutaneous needle biopsy or open surgical biopsy) [6–9]. As the kidney is suited for percutaneous needle biopsy and this method is most commonly chosen, obtained samples are relatively small and insufficient material is obtained in some cases. Therefore, to choose the most appropriate cutoff level in IgG4-RKD, the accumulation of studies focused on the infiltrating number of IgG4+ cells in the kidneys is needed. Our result supported the previously proposed cutoff value of >10/hpf [2]. On the other hand, 15 of 20 patients fulfilled the criterion of IgG4+/IgG+ plasma cell ratio > 40%, while the remaining 5 patients showed a ratio less than or equal to 40%. Thus, the quantitative assessment of infiltrating IgG4-positive plasma cells seems to supplement the IgG4+/IgG+ (CD138+) plasma cell ratio if this ratio is less than or equal to 40%.

 Raissian et al. showed that 25 of 30 patients (83%) had TBM immune complex deposits by immunofluorescence microscopy (IF) or electron microscopy (EM) [2]. In contrast, we found that 47% of patients had C3 deposits in TBM by IF and 13% of them had IgG deposits in TBM by IF. The difference in the frequency of TBM deposits might be due to a population difference, or IF sample size which might be smaller in our study. Although the frequency is different, the fact that more than 40% of patients were shown to have TBM deposits implies a close relationship between TBM deposits and IgG4-RKD. TBM deposits may thus show some immune complex involvement in IgG4-related disease.

Glomerular diseases sometimes concur with tubulointerstitial nephritis in patients with IgG4-related disease [4, 10, 12, 20, 23, 24, 28–30]. These include IgA nephropathy, Henoch-Schönlein purpura nephritis, endocapillary proliferative nephritis, crescentic glomerulonephritis, and membranous glomerulonephritis (MGN). Of these, MGN is the most frequently reported glomerular pathology [10, 12, 30, 33–35]. 

 Interestingly, the first IgG4-RKD case reported by Uchiyama-Tanaka et al. had tubulointerstitial nephritis with MGN, and subepithelial and intramembranous electron-dense deposits disappeared after successful corticosteroid therapy [10]. In contrast, Watson et al. reported a second patient with IgG4-related TIN with MGN, the steroid responsiveness of which differed markedly and whose proteinuria persisted despite 7-months treatment [12]. Although laboratory and immunohistochemical features were not significantly different between IgG4-related TIN with or without glomerular lesions in this study, further studies will be necessary including some focused on the responsiveness to treatment.

 MGN detected during the clinical course of IgG4-RD is classified into two groups based on the presence or absence of simultaneous overlapping of TIN. Cravedi et al. reported a patient with IgG4-RD of the pancreas with salivary gland involvement who developed proteinuria after the cessation of successful steroid therapy [34]. The renal biopsy revealed pure MGN without IgG4+ plasma cell rich TIN. Palmisano et al. also reported a pure MGN development in a patient with IgG4-related chronic periaortitis [35]. These two cases had in common MGN development without IgG4+ plasma cell infiltration in the clinical course of typical IgG4-RD. Although these cases seem to be pure MGN, careful judgment is needed because regional lesion distribution is a feature of IgG4-TIN, and sometimes only unaffected samples are obtained by percutaneous needle biopsy. 

 Although case reports of Henoch-Schönlein purpura (HSP) nephritis associated with IgG4-RD are very rare and only our two cases are so far known [28, 29], occasional development of anaphylactoid purpura in patients with IgG4-RD has been experienced (personal communication). As involvement of an allergic background is commonly presumed in both diseases, we should carefully evaluate the association of HSP with IgG4-RD when IgG4-RD patients have purpura.

 In conclusion, we confirmed that infiltrating IgG4-positive plasma cells >10/hpf and/or IgG4/IgG (CD138)-positive plasma cells >40% was appropriate as an item of the diagnostic criteria for IgG4-RKD. Relatively high frequency of a variety of glomerular lesions concurrent with characteristic IgG4+ plasma-cell-rich lymphoplasmacytic infiltration with fibrosis seemed to show evidence of immune complex involvement in IgG4-related disease. However, as the number of analyzed cases in this study is small and some bias exists in case selection, worldwide study is needed to clarify the accurate frequency of the glomerular lesions in IgG4-RKD and pathophysiological significance of immune deposits in TBM and in the glomerular lesion.

## Figures and Tables

**Table 1:** Clinical and laboratory features of IgG4-related tubulointerstitial nephritis.

Pt. no.	Age/gender	U-Prot	Cr	IgG	IgG4	IgE	CH50	C3	C4	Other organ involvement
1	76/F	—	0.59	2,990	769	267	60	110	27	Sa, Lu
2	70/M	0.26 g/day	0.90	3,496	623	NA	<12	52	2	Pa
3	59/M	—	1.10	2,319	734	542	>66.0	106	24	Sa, Pa, Pr, RP
4	63/M	0.2 g/gCr	1.20	1,756	408	513	51	98	16	Sa, Pa, Lu, Ao
5	58/M	0.2 g/gCr	1.20	3,170	1,204	3,960	<10	33	7	Sa, LN, Lu
6	58/M	—	1.30	1,960	1,280	456	34	81	16	Li, Ne
7	75/M	0.21 g/day	1.34	5,380	587	NA	<14	41	<5	Sa, LN, Lu
8	68/M	0.1 g/day	1.37	2,995	670	2,323	10	41	2	Sa
9	75/M	0.22 g/day	2.34	1,679	890	631	52	81	29	Sa
10	55/M	0.5 g/day	2.10	5,040	1,780	NA	49	74	36	Sa, Pa
11	69/M	0.25 g/day	2.36	4,001	1,340	NA	10	55	2	Pa
12	80/M	0.4 g/day	1.60	4,657	660	NA	<12	35	<1	Pa
13	68/M	—	1.90	3,830	736	NA	3	33	1	Sa, LN
14	79/M	—	0.60	4,756	409	457	8	41	3	Jo
15	69/M	1.0 g/gCr	7.26	4,661	1,120	335	5	10	7	La, Sa, LN, Pa, Lu, Pr
16	72/M	0.22 g/day	0.80	4,359	1,100	537	<12	55	3	LN
17	75/F	3.0 g/gCr	2.25	3,695	486	1,226	2	18	2	Sa, LN, Lu
18	83/M	2.3 g/day	1.48	3,144	944	32.1	16	56	6	—
19	60/M	0.5 g/gCr	1.59	1,952	886	575	56	86	21	La, Sa
20	78/M	1.4 g/day	6.17	3,731	1,860	NA	27.3	57	28	Pa

Note: Conversion factor for Cr: mg/dL to *μ*mol/L, ×88.4.

Abbreviations: Ao: aorta; CH50, serum CH50 (U/mL); Cr: serum creatinine (mg/dL); C3: serum C3 (mg/dL); C4: serum C4 (mg/dL); IgG: serum immunoglobulin G (mg/dL); IgG4: serum immunoglobulin G4 (mg/dL); IgE: serum immunoglobulin E (IU/mL); Jo: joint; La: lacrimal gland; Li: liver; LN: lymph node; Lu: lung; NA: not available; Ne: nerve; Pa: pancreas; Pr: prostate; RP: retroperitoneum; Sa: salivary gland; U-Prot: proteinuria.

**Table 2:** Histologic features of IgG4-related tubulointerstitial nephritis.

		IgG4 IHC		Glomerular	IF TBM	IF TBM	IF TBM	IF GL	IF GL	IF GL	IF GL	EM TBM	EM GL
Pt. no.	Age/gender	(cells per hpf)	IgG4/IgG	Lesion	IgG	C3	C1q	IgG	IgA	C3	C1q
1	76/F	50	81%	−	−	+	−	−	−	−	−	−	−
2	70/M	19	38%	−	NA	NA	NA	NA	NA	NA	NA	+	−
3	59/M	57	54%	−	−	−	−	−	−	−	−	NA	−
4	63/M	37	46%	−	−	+	+	−	−	−	−	−	−
5	58/M	21	81%	−	NA	NA	NA	−	−	−	NA	NA	−
6	58/M	156	77%	−	−	−	−	−	−	−	−	−	−
7	75/M	25	18%	−	−	−	−	NA	NA	NA	NA	+	−
8	68/M	17	40%	−	−	−	−	+	−	+	−	−	−
9	75/M	28	64%	−	+	+	−	−	−	−	−	±	−
10	55/M	49	55%	−	−	−	−	±	−	−	−	−	−
11	69/M	30	51%	−	−	−	−	+	−	−	2+	+	−
12	80/M	10	90%	MPGN	NA	NA	NA	2+	−	2+	+	NA	+
13	68/M	28	38%	IgA GN	−	+	−	−	2+	±	±	NA	NA
14	79/M	42	41%	EC	−	+	+	−	−	−	−	+	−
15	69/M	73	57%	EC	−	−	−	−	−	+	−	−	−
16	72/M	51	58%	HSPN	NA	NA	NA	2+	+	±	−	NA	+
17	75/F	62	40%	HSPN	−	−	−	−	+	2+	−	−	+
18	83/M	25	43%	MGN	+	+	−	+	−	+	−	+	+
19	60/M	68	42%	MGN	−	−	−	3+	−	−	−	−	+
20	78/M	28	45%	MGN	−	+	−	−	−	−	−	−	+

Abbreviations: EC: endocapillary hypercellularity; EM: electron microscopy; GL: glomeruli; hpf: high-power field; HSPN: Henoch-Schönlein purpura nephritis; IF: immunofluorescence; IgA GN: IgA nephropathy; IHC: immunohistochemistry; MGN: membranous glomerulonephritis; MPGN: membranoproliferative glomerulonephritis; NA: not available; Pt.: patient; TBM: tubular basement membranes.

**Table 3:** Laboratory difference between IgG4-TIN patients with glomerular lesions and those without glomerular lesions.

	IgG4-TIN with GL	IgG4-TIN without GL	*P* value
Number of patients	9	11	
Age (years), mean ± SD	73.8 ± 7.2	66.0 ± 7.7	0.036
Serum creatinine (mg/dL)	2.6 ± 2.4	1.4 ± 0.6	0.239
Serum IgG (mg/dL)	3865 ± 903	3162 ± 1251	0.16
Serum IgG4 (mg/dL)	909 ± 434	935 ± 413	0.909
SerumC3 (mg/dL)	43 ± 23	70 ± 27	0.068
Low C4	7/9	5/11	0.197
Low CH50	7/9	5/11	0.197
IgG4 IHC (cells per hpf)	43.0 ± 21.8	44.5 ± 39.4	0.493
IgG4/IgG (%)	50.4 ± 16.5	54.1 ± 20.8	0.518
IF TBM IgG	1/7	1/9	>0.999
IF TBM C3	4/7	3/9	0.615
IF TBM C1q	1/7	1/9	>0.999
EM TBM	2/6	4/9	>0.999

Note: Conversion factor for creatinine: mg/dL to *μ*mol/L, ×88.4.

Abbreviations: EM: electron microscopy; GL: glomerular lesions; hpf: high-power field; IF: immunofluorescence; IHC: immunohistochemistry; TBM: tubular basement membranes; TIN: tubulointerstitial nephritis.

**Table 4:** IgG4-positive plasma-cell-rich ANCA-related vasculitis.

Pt. no.	Age/gender	Diagnosis	PC infiltration	IgG4/hpf	IgG4/CD138 ratio (%)
1	75/F	CSS	++	19	47
2	59/M	mPA	++	22	52
3	79/F	mPA	+++	34	78
4	67/F	RLV	++	19	69

Abbreviations: CSS: Churg-Strauss syndrome; hpf: high-power field; mPA: microscopic polyangiitis; PC: plasma cell; RLV: renal limited vasculitis.
